# Supplementary material for: Malnutrition in Patients with Liver Cirrhosis
Source: Nutrients. 2021 Feb 7;13(2):540. doi: 10.3390/nu13020540 (PMC7915767; doi:10.3390/nu13020540)
Supplement: Supplementary file 1 [file nutrients-13-00540-s001.pdf]

Supplements

Malnutrition in patients with liver cirrhosis

Julia Traub[1§], Lisa Reiss[1§], Benard Aliwa[2], Vanessa Stadlbauer[2]

1) Department of Clinical Medical Nutrition, University Hospital Graz, 8036 Graz, Austria

2) Department of Gastroenterology and Hepatology, Medical University of Graz, 8036 Graz, Austria

§ Contributed equally

* Correspondence: vanessa.stadlbauer@medunigraz.at; Tel.: +43 316-385-82282

Supplementary Table 1: Summary of studies reporting the prevalence of malnutrition, sarcopenia, osteodystrophy and frailty in cirrhosis between 2015 and 2020

Study characteristics	Prevalence of malnutrition	Prevalence of sarcopenia/osteodystrophy	Reference
102 liver transplant candidates, the Netherlands	Not reported	29.4% presarcopenia<br>19.6% sarcopenia	(1)
232, liver transplant candidates, UK	47% high risk on RFH-NTP	Not reported	(2)
109 liver transplant candidates, Sweden	Low fat mass index 15%<br>low fat-free mass index 9%<br>Malnutrition assessment: 5% malnourished, 39% at risk		(3)
315 liver transplant candidates, Canada	60% SGA moderate/severe malnutrition	Low skeletal muscle index 38%	(4)
73 liver transplant candidates, Brazil	60% SGA moderate/severe malnutrition		(5)
117 liver transplant candidates, India	86% SGA moderate/severe malnutrition		(6)
70 liver transplant candidates, Canada	79% SGA moderate/severe malnutrition		(7)

110 liver transplant candidates, Spain	70% malnourished on 4 or more malnutrition scores		(8)
60 cirrhotic inpatients, Thailand	SGA 92% moderate/severe malnutrition	Not reported	(9)
381 cirrhotic inpatients, USA	57% RFH-NTP high risk	Not reported	(10)
120 patients with esophageal varices bleeding, Taiwan	38% high nutrition risk mNUTRIC score		(11)
13.141.324 nationwide inpatient sample, USA	Malnutrition 12.4% in cirrhosis with fracture, 9.6% in cirrhosis without fracture, 5.4 % in patients no cirrhosis with fracture	Not reported	(12)
247 cirrhotic inpatients, India	60% low protein calorie malnutrition score<br>67% malnutrition on body composition analysis	71% reduced handgrip strength	(13)
130 cirrhotic in- and outpatients, Portugal	41%SGA moderate/severe malnutrition		(14)
104 cirrhotic patients, Tunisia	63% SGA moderate/severe malnutrition		(15)
155 cirrhotic patients, China	RFH-NPT 63% at nutritional risk		(16)
170 cirrhotic patients, Greece	SGA 23% moderate/severe malnutrition	Not reported	(17)
122 cirrhotic patients, Pakistan	73% protein calorie malnutrition score<br>61% malnutrition on body composition analysis	Not reported	(18)
84 cirrhotic patients, Germany	51% high risk on RFH-NPT	Not reported	(19)
220 cirrhotic patients, Mexico	39% low phase angle		(20)
42 cirrhotic outpatients, Australia	SGA 40% moderate/severe malnutrition	Not reported	**(21)**
60 cirrhotic outpatients, Nepal	77% malnutrition by protein calorie malnutrition score	Not reported	(22)

406 cirrhotic outpatients, Australia	Not reported	Osteodystrophy 54%	(23)
120 patients with Hep B and C cirrhosis, China	SGA 60% moderate/severe malnutrition	Not reported	(24)
22 cirrhotic outpatients, USA	80% malnutrition by dietician assessment		(25)
118 outpatients, Brazil	Malnutrition 35%	Sarcopenia 17%	(26)
983 cirrhotic outpatients, USA		Liver Frailty Index: 15% frail	(27)
104 patients with chronic hepatitis C with and without cirrhosis, Brazil	Not reported	Low bone mineral density 34.6%<br>low muscle strength 27.9%<br>pre-sarcopenia 14.4%<br>sarcopenia 8.7%<br>sarcopenic obesity 3.8%	(28)
135 patients with chronic hepatitis C (noncirrhotic and compensated cirrhotic), Spain	22% low fat-free mass index<br>24% low phase angle		(29)
352 patients with cirrhosis and 189 patients with chronic hepatitis, India	56%, SGA moderate/severe malnutrition in cirrhosis; 24% in chronic hepatitis	Low handgrip strength 42% in cirrhosis, 18% in chronic hepatitis	(30)

Supplementary Table 2: Percentage of liver cirrhosis patients with inadequate energy intake and percentage reduction in energy intake

Study characteristics	Inadequate energy intake (percentage of patients)	Reference
159 liver cirrhosis patients on waiting list for liver transplant, Brazil	91%	(31)
153 patients with posthepatitic liver cirrhosis, China	73%	(32)
47 hepatitis C virus related cirrhosis, Japan	17%	(33)
73 cirrhosis patients on the waiting list for liver transplantation, Brazil	92%	(34)
81 liver cirrhosis patients, Brazil	100%	(35)
231 liver cirrhosis patients, Australia	56%	(36)

17 liver cirrhosis patients awaiting orthotopic liver transplantation, Australia	25%	(37)
51 cirrhotic candidates for liver transplant, Brazil	67%	(38)
19 liver cirrhosis patiens, Australia	100%	(39)
Study characteristics	Percentage reduction in energy intake	Reference
38 liver cirrhosis patients undergoing liver transplantation, Italy	14%	(40)
17 liver cirrhosis patients before liver transplantation, Brazil	34%	(41)
70 pre-liver transplant cirrhotic patients, Canada	13%	(7)

Supplementary table 3: Observed abnormalities in trace element, mineral and vitamin levels in patients with liver cirrhosis

Element	Liver cirrhosis
Zinc (42, 43)	↓
Selenium (42)	↓
Iron (44)	↓
Magnesium (42, 45)	↓
Copper (42, 46)	↑
Manganese (47)	↑
Vitamin A (48)	↓
Vitamin D (48)	↓
Vitamin E (48)	↓
Vitamin K (48)	↓
Vitamin C (49)	↓
Vitamin B1 (50)	↓
Vitamin B2 (50)	↓
Vitamin B6 (50, 51)	↓
Vitamin B12 (50, 52-54)	↑
Folic acid (53)	↓

1. Oey RC, Aarts P, Erler NS, Metselaar HJ, Lakenman PLM, Riemslag Baas-van der Ree S, et al. Identification and prognostic impact of malnutrition in a population screened for liver transplantation. Clin Nutr ESPEN. 2020;36:36-44.
2. Kalafateli M, Mantzoukis K, Choi Yau Y, Mohammad AO, Arora S, Rodrigues S, et al. Malnutrition and sarcopenia predict post-liver transplantation outcomes independently of the Model for End-stage Liver Disease score. J Cachexia Sarcopenia Muscle. 2017;8(1):113-21.
3. Lindqvist C, Majeed A, Wahlin S. Body composition assessed by dual-energy X-ray absorptiometry predicts early infectious complications after liver transplantation. J Hum Nutr Diet. 2017;30(3):284-91.
4. Moctezuma-Velazquez C, Ebadi M, Bhanji RA, Stirnimann G, Tandon P, Montano-Loza AJ. Limited performance of subjective global assessment compared to computed tomography-determined sarcopenia in predicting adverse clinical outcomes in patients with cirrhosis. Clin Nutr. 2019;38(6):2696-703.
5. Ribeiro HS, Mauricio SF, Antonio da Silva T, de Vasconcelos Generoso S, Lima AS, Toulson Davisson Correia MI. Combined nutritional assessment methods to predict clinical outcomes in patients on the waiting list for liver transplantation. Nutrition. 2018;47:21-6.
6. Yadav SK, Choudhary NS, Saraf N, Saigal S, Goja S, Rastogi A, et al. Nutritional status using subjective global assessment independently predicts outcome of patients waiting for living donor liver transplant. Indian J Gastroenterol. 2017;36(4):275-81.
7. Marr KJ, Shaheen AA, Lam L, Stapleton M, Burak K, Raman M. Nutritional status and the performance of multiple bedside tools for nutrition assessment among patients waiting for liver transplantation: A Canadian experience. Clin Nutr ESPEN. 2017;17:68-74.
8. Garcia-Rodriguez MT, Pertega-Diaz S, Lopez-Calvino B, Pinon-Villar MDC, Otero-Ferreiro A, Suarez-Lopez F, et al. Nomogram and Validity of a Model for Predicting Malnutrition in Patients on Liver Transplant Lists. Dig Dis Sci. 2018;63(7):1952-61.
9. Bunchorntavakul C, Supanun R, Atsawarungruangkit A. Nutritional Status and its Impact on Clinical Outcomes for Patients Admitted to Hospital with Cirrhosis. J Med Assoc Thai. 2016;99 Suppl 2:S47-55.
10. Reuter B, Shaw J, Hanson J, Tate V, Acharya C, Bajaj JS. Nutritional Assessment in Inpatients With Cirrhosis Can Be Improved After Training and Is Associated With Lower Readmissions. Liver Transpl. 2019;25(12):1790-9.
11. Tsai MH, Huang HC, Peng YS, Chen YC, Tian YC, Yang CW, et al. Nutrition Risk Assessment Using the Modified NUTRIC Score in Cirrhotic Patients with Acute Gastroesophageal Variceal Bleeding: Prevalence of High Nutrition Risk and its Independent Prognostic Value. Nutrients. 2019;11(9).
12. Patel A, Silverman S, Baghdadi J, Shah O, Sundaram V. Osteoporotic Fracture Risk and Health Care Burden in Patients With Cirrhosis. J Clin Gastroenterol. 2019;53(7):543-8.
13. Maharshi S, Sharma BC, Sachdeva S, Srivastava S, Sharma P. Efficacy of Nutritional Therapy for Patients With Cirrhosis and Minimal Hepatic Encephalopathy in a Randomized Trial. Clin Gastroenterol Hepatol. 2016;14(3):454-60 e3; quiz e33.
14. Nunes G, Santos CA, Barosa R, Fonseca C, Barata AT, Fonseca J. Outcome and Nutritional Assessment of Chronic Liver Disease Patients Using Anthropometry and Subjective Global Assessment. Arq Gastroenterol. 2017;54(3):225-31.
15. Ennaifer R, Cheikh M, Romdhane H, Sabbagh S, Ben Nejma H, Bougassas W, et al. Does protein energy malnutrition affect the outcome in Tunisian cirrhotic patients? Tunis Med. 2016;94(2):172-6.

16. Wu Y, Zhu Y, Feng Y, Wang R, Yao N, Zhang M, et al. Royal Free Hospital-Nutritional Prioritizing Tool improves the prediction of malnutrition risk outcomes in liver cirrhosis patients compared with Nutritional Risk Screening 2002. Br J Nutr. 2020:1-10.
17. Georgiou A, Papatheodoridis GV, Alexopoulou A, Deutsch M, Vlachogiannakos I, Ioannidou P, et al. Evaluation of the effectiveness of eight screening tools in detecting risk of malnutrition in cirrhotic patients: the KIRRHOS study. Br J Nutr. 2019;122(12):1368-76.
18. Parkash O, Jafri W, Munir SM, Iqbal R. Assessment of malnutrition in patients with liver cirrhosis using protein calorie malnutrition (PCM) score verses bio-electrical impedance analysis (BIA). BMC Res Notes. 2018;11(1):545.
19. Borhofen SM, Gerner C, Lehmann J, Fimmers R, Gortzen J, Hey B, et al. The Royal Free Hospital-Nutritional Prioritizing Tool Is an Independent Predictor of Deterioration of Liver Function and Survival in Cirrhosis. Dig Dis Sci. 2016;61(6):1735-43.
20. Ruiz-Margain A, Macias-Rodriguez RU, Ampuero J, Cubero FJ, Chi-Cervera L, Rios-Torres SL, et al. Low phase angle is associated with the development of hepatic encephalopathy in patients with cirrhosis. World J Gastroenterol. 2016;22(45):10064-70.
21. Luong R, Kim M, Lee A, Carey S. Assessing nutritional status in a cohort of liver cirrhosis outpatients: A prospective cross-sectional study. Nutr Health. 2020;26(1):19-25.
22. Khadka D, Karki B, Thapa S, Khanal A, Shrestha R, Bhandary S, et al. Prevalence of Malnutrition in Patients with Liver Cirrhosis in A Tertiary Care Hospital. JNMA J Nepal Med Assoc. 2019;57(218):229-33.
23. Chinnaratha MA, Chaudhary S, Doogue M, McCormick RJ, Woodman RJ, Wigg AJ. Prevalence of hepatic osteodystrophy and vitamin D deficiency in cirrhosis. Intern Med J. 2015;45(12):1230-5.
24. Yao J, Chang L, Yuan L, Duan Z. Nutrition status and small intestinal bacterial overgrowth in patients with virus-related cirrhosis. Asia Pac J Clin Nutr. 2016;25(2):283-91.
25. Booi AN, Menendez J, Norton HJ, Anderson WE, Ellis AC. Validation of a Screening Tool to Identify Undernutrition in Ambulatory Patients With Liver Cirrhosis. Nutr Clin Pract. 2015;30(5):683-9.
26. Zambrano DN, Xiao J, Prado CM, Gonzalez MC. Patient-Generated Subjective Global Assessment and Computed Tomography in the assessment of malnutrition and sarcopenia in patients with cirrhosis: Is there any association? Clin Nutr. 2020;39(5):1535-40.
27. Lai JC, Dodge JL, McCulloch CE, Covinsky KE, Singer JP. Frailty and the Burden of Concurrent and Incident Disability in Patients With Cirrhosis: A Prospective Cohort Study. Hepatol Commun. 2020;4(1):126-33.
28. Bering T, Diniz KGD, Coelho MPP, Vieira DA, Soares MMS, Kakehasi AM, et al. Association between pre-sarcopenia, sarcopenia, and bone mineral density in patients with chronic hepatitis C. J Cachexia Sarcopenia Muscle. 2018;9(2):255-68.
29. Bering T, Diniz KGD, Coelho MPP, de Souza ACM, de Melo LF, Vieira DA, et al. Bioelectrical Impedance Analysis-Derived Measurements in Chronic Hepatitis C: Clinical Relevance of Fat-Free Mass and Phase Angle Evaluation. Nutr Clin Pract. 2018;33(2):238-46.
30. Sharma P, Rauf A, Matin A, Agarwal R, Tyagi P, Arora A. Handgrip Strength as an Important Bed Side Tool to Assess Malnutrition in Patient with Liver Disease. J Clin Exp Hepatol. 2017;7(1):16-22.
31. Ferreira LG, Anastacio LR, Lima AS, Correia MI. [Malnutrition and inadequate food intake of patients in the waiting list for liver transplant]. Rev Assoc Med Bras (1992). 2009;55(4):389-93.

32. Meng QH, Yu HW, Li J, Wang JH, Ni MM, Feng YM, et al. Inadequate nutritional intake and protein-energy malnutrition involved in acute and chronic viral hepatitis Chinese patients especially in cirrhosis patients. Hepatogastroenterology. 2010;57(101):845-51.
33. Yasutake K, Bekki M, Ichinose M, Ikemoto M, Fujino T, Ryu T, et al. Assessing current nutritional status of patients with HCV-related liver cirrhosis in the compensated stage. Asia Pac J Clin Nutr. 2012;21(3):400-5.
34. Ferreira LG, Ferreira Martins AI, Cunha CE, Anastacio LR, Lima AS, Correia MI. Negative energy balance secondary to inadequate dietary intake of patients on the waiting list for liver transplantation. Nutrition. 2013;29(10):1252-8.
35. Ferreira LG, Santos LF, Silva TR, Anastacio LR, Lima AS, Correia MI. Hyper- and hypometabolism are not related to nutritional status of patients on the waiting list for liver transplantation. Clin Nutr. 2014;33(5):754-60.
36. Huynh DK, Selvanderan SP, Harley HA, Holloway RH, Nguyen NQ. Nutritional care in hospitalized patients with chronic liver disease. World J Gastroenterol. 2015;21(45):12835-42.
37. McCoy SM, Campbell KL, Lassemillante AM, Wallen MP, Fawcett J, Jarrett M, et al. Changes in dietary patterns and body composition within 12 months of liver transplantation. Hepatobiliary Surg Nutr. 2017;6(5):317-26.
38. Viana ACC, Maia FMM, Carvalho NS, Morais SR, Bezerra AN, Mendonca PDS, et al. Correlation between nutritional assessment and oxidative stress in candidates for liver transplant. Einstein (Sao Paulo). 2020;18:eAO4039.
39. Chapman B, Gow P, Sinclair M, Hanrahan T, Angus P, McClure T, et al. Continuous terlipressin infusion is associated with improved diet intake and muscle strength in patients awaiting liver transplant. JHEP Rep. 2019;1(2):107-13.
40. Merli M, Giusto M, Gentili F, Novelli G, Ferretti G, Riggio O, et al. Nutritional status: its influence on the outcome of patients undergoing liver transplantation. Liver Int. 2010;30(2):208-14.
41. Ferreira LG, Santos LF, Anastacio LR, Lima AS, Correia MI. Resting energy expenditure, body composition, and dietary intake: a longitudinal study before and after liver transplantation. Transplantation. 2013;96(6):579-85.
42. Nangliya V, Sharma A, Yadav D, Sunder S, Nijhawan S, Mishra S. Study of trace elements in liver cirrhosis patients and their role in prognosis of disease. Biol Trace Elem Res. 2015;165(1):35-40.
43. Solis-Herruzo J, De Cuenca B, Munoz-Rivero MC. Intestinal zinc absorption in cirrhotic patients. Z Gastroenterol. 1989;27(6):335-8.
44. Buyukasik NS, Nadir I, Akin FE, Cakal B, Kav T, Ersoy O, et al. Serum iron parameters in cirrhosis and chronic hepatitis: detailed description. Turk J Gastroenterol. 2011;22(6):606-11.
45. Pasqualetti P, Casale R, Colantonio D, Di Lauro G, Festuccia V, Natali L, et al. [Serum levels of magnesium in hepatic cirrhosis]. Quad Sclavo Diagn. 1987;23(1):12-7.
46. Jurczyk K, Wawrzynowicz-Syczewska M, Boron-Kaczmarska A, Sych Z. Serum iron parameters in patients with alcoholic and chronic cirrhosis and hepatitis. Med Sci Monit. 2001;7(5):962-5.
47. Agarwal A, Avarebeel S, Choudhary NS, Goudar M, Tejaswini CJ. Correlation of Trace Elements in Patients of Chronic Liver Disease with Respect to Child- Turcotte- Pugh Scoring System. J Clin Diagn Res. 2017;11(9):OC25-OC8.
48. Teriaky A, Mosli M, Chandok N, Al-Judaibi B, Marotta P, Qumosani K. Prevalence of fat-soluble vitamin (A, D, and E) and zinc deficiency in patients with cirrhosis being assessed for liver transplantation. Acta Gastroenterol Belg. 2017;80(2):237-41.

49. Beattie AD, Sherlock S. Ascorbic acid deficiency in liver disease. Gut. 1976;17(8):571-5.
50. Majumdar SK, Shaw GK, O'Gorman P, Aps EJ, Offerman EL, Thomson AD. Blood vitamin status (B1, B2, B6, folic acid and B12) in patients with alcoholic liver disease. Int J Vitam Nutr Res. 1982;52(3):266-71.
51. Labadarios D, Rossouw JE, McConnell JB, Davis M, Williams R. Vitamin B6 deficiency in chronic liver disease--evidence for increased degradation of pyridoxal-5'-phosphate. Gut. 1977;18(1):23-7.
52. Sugihara T, Koda M, Okamoto T, Miyoshi K, Matono T, Oyama K, et al. Falsely Elevated Serum Vitamin B12 Levels Were Associated with the Severity and Prognosis of Chronic Viral Liver Disease. Yonago Acta Med. 2017;60(1):31-9.
53. Zhou YJ, Liang MY, Zhang XQ. [Changes in serum folic acid and vitamin B12 levels in liver cirrhosis and its clinical significance]. Zhonghua Nei Ke Za Zhi. 1992;30(10):625-7, 58.
54. Areekul S, Panatampon P, Doungbarn J. Vitamin B12 and vitamin B12 binding proteins in liver diseases. Southeast Asian J Trop Med Public Health. 1977;8(3):322-8.
